# Extracellular vesicles in rheumatoid arthritis: emerging roles in progression, diagnosis, and therapeutic development

**DOI:** 10.3389/fimmu.2026.1748591

**Published:** 2026-02-10

**Authors:** Qisong Liu, Lili He, Xiaomin Wu, Xiaohua Pan

**Affiliations:** Institute of Clinical Translation and Regenerative Medicine, The Second Affiliated Hospital of Shenzhen University (The People’s Hospital of Baoan Shenzhen), Shenzhen, China

**Keywords:** rheumatoid arthritis, extracellular vesicle, biomarker, cell-cell communication, mesenchymal stem cell

## Abstract

Rheumatoid arthritis (RA) is a chronic autoimmune disorder that is pathologically defined by persistent synovitis and systemic inflammation. Currently, the clinical diagnosis and management of RA remain challenging, particularly with respect to early detection, the treatment of refractory cases, and ensuring long-term medication safety. Therefore, it is imperative to deepen our understanding of RA pathogenesis, identify specific biomarkers, and develop innovative therapeutic strategies. This review summarizes the roles and recent advances concerning extracellular vesicles (EVs) in RA progression, diagnosis, and therapeutic development. Research indicates that during RA development, joint-resident cells, immune cells, and relevant body fluids form a complex network in which EV-mediated signaling amplifies inflammatory responses and exacerbates tissue damage. Moreover, studies have shown that EVs isolated from synovial fluid and the circulation of RA patients exhibit significantly altered expression profiles, morphology, or subtype composition. These alterations are closely associated with disease activity, underscoring their potential as diagnostic biomarkers and tools for monitoring disease severity. Regarding therapy, EVs derived from diverse cellular sources have demonstrated promising therapeutic potential in RA. They not only carry bioactive molecules that can modulate RA-associated cells, but also serve as engineered delivery vehicles for targeted therapeutic interventions. In summary, EVs play multifaceted roles in the progression, diagnosis, and treatment of RA. Future research should focus on translating EV-related discoveries into clinical applications, thereby supporting the development of novel strategies for the precise diagnosis and management of RA.

## Introduction

Exosomes, first identified in the 1980s during reticulocyte maturation, were long regarded as cellular “garbage bags” for metabolic waste and unwanted membrane proteins (1). However, research over the past two decades has overturned this view. We now recognize exosomes as extracellular vesicles (EVs) of 30–150 nm in diameter, originating from the endosomal system and released via plasma membrane fusion (2). Other EVs include microvesicles (MVs) and apoptotic bodies (ApoBDs), formed respectively by membrane budding and apoptosis. Due to limitations in isolation techniques, small MVs and exosomes are often co-isolated; we refer to this mixture as “EVs”. Larger MVs, separable by lower-speed centrifugation, are specifically termed “MVs”.

EVs function primarily as sophisticated information carriers that mediate intercellular communication (3). They are delivered throughout the body via bodily fluids and internalized by recipient cells. This process transfers their functional molecular cargo to target cells and modulate cellular physiology. In disease contexts, this transfer mechanism can become a critical contributor to pathogenesis (4). For example, in autoimmune disorders, EVs derived from compromised cells may be functionally programmed to deliver aberrant signals and pro-inflammatory mediators, thereby exacerbating inflammatory responses and hastening disease advancement (5). By forming an efficient intercellular delivery system, EVs thus play an essential role in both the onset and progression of diseases (4, 5).

EVs are abundant in various bodily fluids, including blood, synovial fluid, and urine. Under pathological conditions, these EVs carry molecular information from their parent cells, making them highly promising candidates as biomarkers for disease diagnosis (6, 7). Quantifying EV abundance, morphology, or profiling their protein and nucleic-acid cargo, enables real-time tracking of disease progression (8–11). This liquid-biopsy strategy delivers a powerful, minimally invasive alternative to conventional tissue sampling, offering repeatability and dynamic monitoring capacity (12, 13). By enabling earlier diagnosis, precise patient stratification, prognostic assessment, and real-time monitoring of therapeutic response, EV-based liquid biopsies are accelerating the translation of precision medicine into clinical practice (14).

Beyond diagnostics, EVs are emerging as versatile therapeutic tools due to their dual functional capabilities. Firstly, EVs can inherit key biological traits from their parent cells (15). For example, mesenchymal stem cell-derived EVs (MSC-EVs) possess immunomodulatory properties similar to those of MSCs, presenting a promising cell-free alternative to conventional cell-based therapies (16). Secondly, EVs can be engineered as drug delivery vehicles (17). Their natural biocompatibility, low immunogenicity, ability to cross the blood-barrier, and superior targeting capacity make them an ideal platform for therapeutic delivery. By leveraging these advantages, clinical trials have been launched, advancing EV-based therapy toward clinical translation (18).

Together, EVs have demonstrated significant value in elucidating disease mechanisms, advancing diagnostic technologies, and informing therapeutic strategies. Focusing on rheumatoid arthritis (RA), a complex autoimmune disorder, this review systematically examines the role of EVs across disease progression, diagnosis, and therapeutic innovation.

## EV-mediated cell-cell communication in RA progression

**Figure 1:**
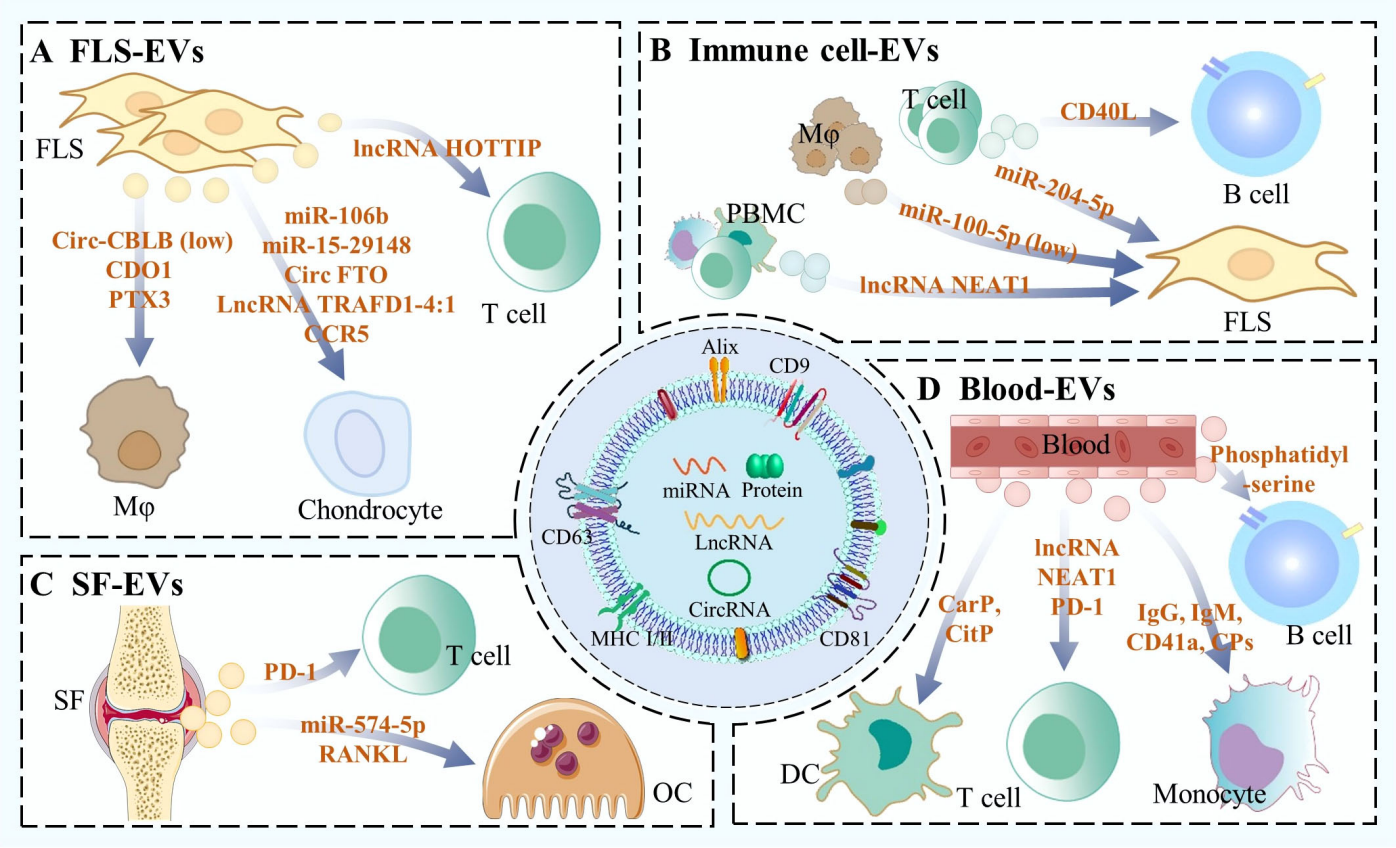
EV mediated cell-cell communication in RA progression. During the progression of RA, EVs originating from FLS, immune cells, synovial fluid, and the circulatory system regulate RA-associated cells via the transfer of RNAs, proteins and lipids, thereby modulating the course of the disease.

In the pathological context of RA, EVs serve as crucial mediators of intercellular communication. During disease progression, EVs derived from fibroblast-like synoviocytes (FLS) and immune cells, as well as those present in synovial fluid and blood, can interact with other cells. These interactions regulate inflammatory responses, cartilage degradation, and bone erosion, thereby influencing disease progression and forming a complex regulatory network (**Figure 1**).

### The role of FLS-derived EVs in the pathogenesis of RA

FLS are critically involved in RA as central aggressors (19). Their excessive proliferation, enhanced anti-apoptotic capacity, and transition to an invasive, activated state collectively promote synovial hyperplasia and the formation of an invasive pannus, thereby driving RA pathogenesis (20). Beyond these direct effects, FLS also exert paracrine control over stromal and immune cells, a process in which EVs function as crucial mediators (21).

Chondrocytes serve as the primary cellular targets of FLS-derived EVs (FLS-EVs) in RA. For instance, EVs from RA patient-derived FLS (RA-FLS-EVs) deliver miR-106b to suppress the expression of pyruvate dehydrogenase kinase 4 (PDK4) in chondrocytes, impairing cartilage repair (22). Another study demonstrated that RA-FLS-EVs promote chondrocyte apoptosis by targeting and inhibiting the anti-apoptotic gene cytokine-induced anti-apoptosis inhibitor 1 (CIAPIN1), mediated by exosomal miRNA 15-29148 (23). Beyond miRNAs, RA-FLS-EVs carry regulatory circular RNAs (circRNAs) and long non-coding RNAs (lncRNAs). CircFTO, for example, facilitates the m6A-dependent degradation of SRY-Box transcription factor 9 (SOX9) messenger RNA (mRNA), thus impairing chondrocyte function (24). Similarly, lnc-TRAFD1-4:1 from RA-FLS-EVs acts as a competitive endogenous RNA for miR-27a-3p, leading to increased chemokine (C-X-C motif) ligand 1 (CXCL1) expression and aggravating cartilage damage (25). Additionally, proteins carried by RA-FLS-EVs, such as C-C chemokine receptor type 5 (CCR5), can activate the nuclear factor kappa-B (NF-κB) signaling pathway, further promoting chondrocyte apoptosis and extracellular matrix (ECM) degradation (26).

RA-FLS-EVs also play a crucial role in regulating immune cells. Macrophages, key drivers of synovial inflammation, are polarized by RA-FLS-EVs (27). For instance, circ-CBLB carried by FLS-EVs modulates macrophage polarization; however, under inflammatory conditions, it is degraded via Wilms’ tumor 1-associating protein (WTAP)-mediated m6A modification, shifting macrophages toward the pro-inflammatory M1 phenotype (28). Additionally, RA-FLS-EVs carrying cysteine dioxygenase 1 (CDO1) and pentraxin 3 (PTX3) amplify local inflammation by promoting M1 polarization and macrophage migration, respectively (29, 30). Beyond macrophages, T cells, central to RA autoimmune responses, are also regulated by FLS-EVs (31). It is reported that RA-FLS-EVs disrupt the T helper 17 (Th17)/regulatory T cells (Tregs) balance, promoting inflammation and joint damage (32). This effect is mediated by the exosomal lncRNA HOTTIP (HOXA transcript at the distal tip), which sequesters miR-1908-5p to relieve its suppression of signal transducer and activator of transcription 3 (STAT3).

### The role of immune cell-derived EVs in the pathogenesis of RA

In RA pathogenesis, various immune cells orchestrate core pathological events including chronic inflammation, synovial tissue hyperplasia, and joint structural damage (33). These events are driven by inflammatory reactions, such as the secretion of cytokines and antibodies. Moreover, immune cells further contribute to disease progression by releasing EVs, which regulate the function of RA-associated cells.

During RA progression, T cells secret EVs to modulate the functions of B cells and FLS. A study by Lu et al. demonstrated that EVs derived from T follicular helper (Tfh) cells carry high surface CD40 ligand (CD40L), which directly promotes B cell differentiation and antibody production (34). In both collagen-induced arthritis (CIA) models and RA patients, levels of these Tfh-EVs and their surface CD40L were significantly elevated and positively correlated with disease severity. In another study, Wu et al. found that EVs derived from plasma T lymphocyte of RA patients could suppress the proliferation, invasion, and inflammatory factor secretion of synovial cells, indicating a protective role in RA (35). These EVs are enriched with miR-204-5p, which target genes such as CRK like proto-oncogene adaptor protein (CRKL) and angiopoietin 1 (ANGPT1) under inflammatory conditions.

Macrophages can also release EVs to regulate FLS during RA progression. Liu et al. found that macrophage-derived EVs (Mφ-EVs) contained significantly reduced levels of miR-100-5p in an arthritis mouse model (36). Upon uptake by FLS, these miR-100-5p-deficient EVs alleviated the inhibition of mammalian target of rapamycin (mTOR) signaling, thereby promoting synovial cell proliferation and enhancing the secretion of inflammatory factors. This process exacerbates joint inflammation and destruction. Importantly, restoring miR-100-5p expression reversed these pro-inflammatory effects and alleviated arthritic symptoms.

Furthermore, EVs derived from peripheral blood mononuclear cells (PBMCs) have been demonstrated to regulate FLS function and drive RA progression. A study by Rao et al. reported that PBMC-derived EVs from RA patients carry significantly elevated levels of lncRNA NEAT1 (nuclear enriched abundant transcript 1) (37). Upon internalization by FLS, these EVs suppress miR-23a expression and upregulate murine double minute-2 (MDM2) protein. This leads to sirtuin 6 (SIRT6) ubiquitination and degradation, activates the NF-κB signaling pathway, and ultimately enhances FLS proliferation and the secretion of inflammatory factors, thereby aggravating RA pathogenesis.

### The role of synovial fluid-derived EVs in the pathogenesis of RA

In RA, synovial fluid undergoes a critical shift from its physiological role as a joint lubricant to a pathogenic microenvironment that actively drives disease progression (38). In patients, it is enriched with pro-inflammatory factors, creating a self-sustaining inflammatory milieu that exacerbates pathology (39). Moreover, synovial fluid contains abundant EVs derived from pathologically activated cells. These EVs can package and deliver bioactive molecules, modulating the behavior of local stromal and immune cells and ultimately driving articular destruction.

In RA, synovial fluid-derived EVs (SF-EVs) act as integral components of the inflammatory network. Studies indicate that in RA patients, SF-EVs isolated from highly inflamed joints contain 3.5-fold more total miRNA than those from low-inflammation joints, with 78 differentially expressed miRNAs identified (40). Of these, 49 miRNAs were upregulated and their target genes were enriched in pro-inflammatory processes, including cytokine-mediated signaling pathways. In another study, Greisen et al. showed that RA SF-EVs display the co-inhibitory receptor programmed death 1 (PD-1). These PD-1-carrying EVs may promote T cell exhaustion and thereby contribute to RA progression (41).

In RA, joint destruction and associated disability are closely linked to osteoclasts (OCs) activities (42, 43). The abnormal activation and excessive differentiation of OCs promote increased bone resorption, leading to subchondral bone erosion, progressive bone loss, and ultimately irreversible joint damage. Notably, SF-EVs contribute to OC dysregulation in the pathogenesis of RA. Hegewald et al. reported that SF-EVs from RA patient significantly promote OC differentiation via their highly expressed miR-574-5p (44). Mechanistically, this miRNA acts as an endogenous ligand for Toll-like receptors 7/8 (TLR7/8), directly binding and activating TLR7/8 signaling to drive OC differentiation. In another study, Song et al. demonstrated that RA SF-EVs could effectively induce CD14^+^ monocytes to differentiate into OCs even without exogenous macrophage colony-stimulating factor (M-CSF) and receptor activator for nuclear factor-κ B ligand (RANKL) (45). This effect is largely attributed to the markedly higher expression of membrane-bound and intravesicular RANKL in RA SF-EVs compared to those from other inflammatory arthritides.

### The role of blood-derived EVs in the pathogenesis of RA

The circulatory system drives RA progression by shuttling activated immune cells and inflammatory mediators (46). Meanwhile, blood carries an abundance of EVs with regulatory functions, which are capable of modulating the activity of immune cells involved in RA pathogenesis (47). In this section, we review the mechanisms by which blood-derived EVs regulate immune cells in RA.

B cells contribute to RA through autoantibody production, antigen presentation and cytokine secretion (48). In these processes, blood-derived EVs exert regulatory functions. A study by Rincón Arévalo et al. demonstrated that medium/large extracellular vesicles (m/lEVs) isolated from the plasma of RA patient markedly suppress early B cell responses triggered by B cell receptor triggering, in a manner partially dependent on phosphatidylserine binding (49). However, in the presence of other immune cells, including T cells and monocyte-derived macrophages, m/lEVs exhibit opposite effects. Specifically, m/lEVs promoted high antibody levels in B cells cultured under T cell-dependent stimulation for 7 days. Moreover, when B cells were co-cultured with autologous monocyte-derived macrophages that had been pre-exposed to m/lEVs, an increased frequency of CD69^+^ B cells was observed. These findings suggest that while m/lEVs can directly suppress early B cell responses, this regulatory function may be compromised in patients with RA, as the vesicles indirectly enhance B cell activity though interactions with other immune cells.

Monocytes are central inflammatory mediators in seropositive RA primary through the production of pro-inflammatory cytokines (50). This process is modulated by blood-derived EVs. Burbano et al. demonstrated that EVs isolated from the plasma of seropositive RA patients are highly enriched with immunoglobulin G (IgG), immunoglobulin M (IgM), and citrullinated peptides (CPs) (51). These EVs are efficiently internalized by monocytes, inducing a robust pro-inflammatory response characterized by elevated secretion of interleukin 1β (IL-1β), interleukin 6 (IL-6), and tumor necrosis factor α (TNF-α). This EV-monocyte interaction effectively recapitulates key features of the systemic inflammation observed in seropositive RA, suggesting it may constitute an important mechanism underlying the more severe disease phenotype in this patient subset.

In the pathogenesis of RA, a pivotal role is played by CD4^+^ T cell activity and their differentiation into Th17 subsets, processes that are partially modulated by blood-derived EVs (52). For example, Liu et al. showed that serum-derived EVs promote RA progression by enhancing CD4^+^ T cell proliferation, inhibiting apoptosis, and facilitating Th17 differentiation and migration (53). Mechanistically, these EVs deliver the lncRNA NEAT1, which sequesters miR-144-3p, thereby relieving the suppression of Rho-associated protein kinase 2 (ROCK2) and subsequently activates the Wnt pathway. In another study, Greisen et al. reported that plasma-derived EVs from RA patients could transfer surface PD-1 to PD-1–negative T cells, inducing ectopic PD-1 expression (25). This transfer, together with dysregulated miRNA-mediated control, exacerbates T cell dysfunction and exhaustion, contributing to the chronic inflammation state in RA.

Dendritic cells (DCs) serve as central regulators in the initiation and maintenance of autoimmune responses in RA. They exert their function through autoantigen presentation, effector T cell activation, and pro-inflammatory cytokine production (54). The functionality of DCs is influenced by blood-derived EVs. Research by Buttari et al. demonstrated that plasma-derived MVs from RA patients potently activate DCs by delivering citrullinated and carbamylated proteins (CitP and CarP) (55). This MV-mediated activation triggers DC maturation, stimulates the mitogen-activated protein kinases (MAPK) and NF-κB signaling pathways, and enhances the secretion of pro-inflammatory cytokines. Consequently, DCs acquire robust pro-inflammatory and T cell-stimulatory capacities. In this way, plasma-derived MVs act as critical mediators of aberrant DC activation and the amplification of autoimmunity in RA.

## EV as biomarker for the diagnosis of RA

EVs have emerged as promising biomarkers in liquid biopsy due to their unique advantages for disease diagnosis (13). In RA, both blood and synovial fluid have been extensively studied as key sources of biomarker-rich EVs. Specific EV characteristics, including molecular cargo, morphological features, and the concentration of particular EV subtypes, show potential not only as diagnostic biomarkers for RA, but also as indicators of clinical parameters like disease activity (**Table 1**).

**Table 1:** Promising EV-based markers for RA diagnosis.

EV source	EV subtype	Key biomarker findings in RA	Significance	Ref
Synovial fluid	EV	↑ PZP & stromelysin-1 (vs. OA)	Exosomal PZP level in RA SF higher than in OA (*p* < 0.05)	(56)
	EV	Altered EV morphology: “gel-out” pattern (RA) vs. “gel-in” (HC)	Not discussed	(58)
	EV	↓ EV & HA particle fluorescence intensity (vs. OA)	Discriminant analysis based on EV/HA parameters correctly classified 100% of samples	(59)
	large HA-containing EV (HA-EVs)	↓HA-EV count & percentage (vs. OA)	In knees SF, HA–EV count and percentage were lower in RA than OA (*p* = 0.023–0.027)	(60)
	MP	↑ Proportion of Annexin V^+^ MP & leukocyte-/granulocyte-/monocyte-/T cell-derived MP (vs. OA)	*p* < 0.001	(61)
Serum	EV	↑ miR-451a & miR-25-3p (vs. HC)	Combined with sTWEAK, achieved AUC 0.983, 100% specificity, 85.7% sensitivity in diagnosing early RA	(62)
	EV	↑ Lnc RNA TCONS_I2_00013502; ↓ ENST00000363624 (vs. HC)	Combined with anti-CCP yielded AUC 0.966, sensitivity 92%, specificity 84%	(65)
	EV	↓ CCL5 & MPIG6B & PFKP mRNA (vs. HC, OA)	Three-gene panel differentiated RA from OA with AUC 0.845	(66)
	EV	↑ CD14 & HLA-DR (vs. HC, OA)	Relative fluorescence index higher in RA (CD14: *p* < 0.05; HLA-DR: *p* < 0.01)	(67)
	EV	↑ AA; ↓LYVE-1 (non-clinical remission vs. clinical remission RA)	Exosomal AA higher and LYVE-1 lower in non-remission group (*p* = 0.001 and *p* = 0.01, respectively)	(70)
Plasma	EV	↑ miR-335-5p (vs. HC)	Positively correlated with anti-CCP levels (R = 0.62, *p* = 0.03)	(63)
	EV	↓ miR-144-3p & miR-30b-5p (vs. HC)	Both miRNAs negatively correlated with DAS28, anti-CCP, and RF	(64)
	EV	Altered EV surface markers: ↑ CD9^+^ or CD81^+^ single-positive EVs; ↓ CD81^+^/CD9^+^ double-positive EVs (vs. HC)	Significant shift in EV subpopulations (*p* = 0.04 for single-positive; *p* = 0.002 for double-positive reduction)	(68)
	MV	↑ CitP on MVs (vs. HC); Citrullinated levels correlates with disease severity	*p* < 0.001; CitP level correlated with DAS28, CDAI and SDAI (*p* = 0.0095; *p* = 0.0091; *p* = 0.0041)	(69)
	MP	↑ Proportion of Annexin V^+^ MP & platelet-derived MP & leukocyte-derived MP (vs. OA, HC)	*p* < 0.001	(61)
	EV	Presence of IgM-RF^+^ EVs associates with higher disease activity in seropositive RA	Correlated with DAS28, VAS and ESR (*p* < 0.05, *p* < 0.001, *p* < 0.01, respectively)	(72)
Blood	CD4+ T cell-derived EV	↑ DPYSL3; ↓ PSME1 (vs. HC)	DPYSL3: *p* = 0.0098; PSME1: *p* = 0.0291	(71)

### SF-EVs as diagnostic candidates for RA

As the primary site of RA pathology, synovial fluid contains components closely linked to disease progression. Among these, the molecular cargo, quantity, and morphology of SF-EVs directly reflect disease status. Thus, systematic analysis of EV-associated changes could help identify diagnostically relevant features, offering new liquid biopsy-based indicators for early detection, patient stratification, and disease monitoring in RA.

Molecular cargo, including proteins and nucleic acids, is altered in SF-EV in RA, highlighting its potential as a source of diagnostic biomarkers. Huang et al. performed the first systematic comparison of SF-EV protein expression profiles across patients with RA, axial spondyloarthritis (axSpA), gout, and osteoarthritis (OA) (56). The study identified several highly expressed proteins specific to RA, including pregnancy zone protein (PZP) and stromelysin-1, suggesting new directions for biomarker discovery in RA. Beyond differentiating among different joint diseases, SF-EV proteins can also assist in patient stratification. Foers et al. reported that, compared to SF-EVs from RA joint with low inflammation, vesicles from highly inflamed RA joints were more abundant, smaller in size, and exhibited a distinct protein profile (57).

Synovial fluid is rich in macromolecules, which can bind to EVs, resulting in morphological features that differ from those of conventional EVs. In RA, compositional alteration of these macromolecules further influence EV morphology. For example, Filali et al. reported that under RA conditions, SF-EVs shift from the multilamellar “gel-in” vesicles observed in healthy states to “gel-out” vesicles (58). In another study, Mustonen et al. used confocal microscopy to quantify hyaluronic acid (HA) particles, SF-EVs and large HA-containing EVs (HA-EVs) in human knee synovial fluid (59). They observed significantly lower fluorescence intensities for both EVs and HA particles in RA patients compared with OA patients and healthy controls. Subsequent work by the same research group further demonstrated that the count and proportion of HA-EVs were reduced in knee synovial fluid from RA patients relative to OA patients (60). Notably, HA-EV levels showed a positive correlation with pain intensity, independent of age and obesity. Collectively, these findings indicate that SF-EV morphology reflect disease status, largely driven by changes of macromolecule composition in synovial fluid.

Under RA pathological conditions, the activity of disease-associated cells varies, leading to altered corresponding changes in the EV subtypes derived from these cells within the synovial fluid. Michael et al. reported that the proportion of annexin V^+^ microparticles (MPs) and MPs originating from leukocyte-derived subsets (including granulocytes, monocytes, and T cells) was significantly higher in the synovial fluid of RA patients compared to those with OA (61). Notably, granulocyte-derived MPs were specifically elevated in anti-cyclic citrullinated peptide antibodies (ACPA)-positive RA patients and in those with longer disease duration. Moreover, their levels correlated positively with ACPA titers. These observations suggest that the abundance of specific EV subtypes may serve as diagnostic biomarkers for RA, mirroring disease progression.

### Circulating EVs as diagnostic candidates for RA

As a systemic autoimmune disease, RA progressively alters circulating biological factors. Clinically, blood markers such as rheumatoid factor (RF) and ACPA are routinely measured for diagnosis. Concurrently, circulating EVs show strong promise as novel RA biomarkers. Their cargo of nucleic acids and proteins furnishes more comprehensive, disease-specific information than conventional assays, enabling earlier and more sensitive detection and monitoring.

Nucleic acids represent crucial components within EVs, forming disease-specific “molecular fingerprint” under pathological conditions. In RA, several EV-derived nucleic acid types have been identified as relevant biomarkers. For example, Rodríguez-Muguruza et al. demonstrated that serum exosomal miR-451a and miR-25-3p serve as high-value biomarkers for early RA diagnosis (62). When combined with the inflammatory factor soluble TNF-related weak inducer of apoptosis (sTWEAK), their diagnostic performance exceeds that of the conventional marker ACPA. Similarly, Yu et al. reported significant upregulation of miR-335-5p and miR-483-5p in plasma EVs from RA patients, with miR-335-5p positively correlating with anti-cyclic citrullinated peptide (anti-CCP) levels (63). In another study, Lu et al. observed decreased expression of plasma exosomal miR-144-3p and miR-30b-5p in RA, both of which negatively correlated with disease activity (64). The combination of these two miRNAs achieved an area under the curve (AUC) of 0.814 for diagnosis. Beyond miRNAs, lncRNAs also show diagnostic potential. Wu et al. identified aberrant expression of two serum exosomal lncRNAs, TCONS_12_00013502 and ENST00000363624, in RA patients (65). The combination of these two exosomal markers together with anti-CCP achieved an AUC of 0.966 for diagnosis. This composite biomarker panel further exhibited a diagnostic specificity of 84% and a sensitivity of 92%. Furthermore, EV-associated mRNAs are emerging as promising biomarkers. Xue et al. reported abnormal expression of C-C motif chemokine ligand 5 (CCL5), megakaryocyte and platelet inhibitory receptor G6b (MPIG6B), and phosphofructokinase platelet type (PFKP) mRNAs in serum EVs from RA patients, with a three-mRNA panel achieving an AUC of 0.845 in discriminating RA from OA (66). In summary, circulating EV-derived nucleic acids (spanning miRNAs, lncRNAs, and mRNAs) exhibit substantial clinical potential as sensitive and specific biomarkers for RA diagnosis and disease activity assessment.

Beyond nucleic acids, exosomal proteins carry substantial biological information and serve as valuable resources for disease monitoring. Membrane proteins are of particular interest due to their accessibility for detection using techniques such as flow cytometry. Gomez et al. reported elevated levels of CD14 and human leukocyte antigen DR (HLA-DR) on serum EVs from RA patients, which helped distinguish RA from other joint diseases (67). Rydland et al. identified abnormal expression patterns of tetraspanins CD9 and CD81 in EVs from RA patients, which were associated with response to methotrexate (MTX) treatment (68). Ucci et al. found that plasma MVs are enriched with CitP, the levels of which positively correlate with disease activity (69). Beyond surface markers, intravesicular proteins such as serum amyloid A (AA) and lymphatic vessel endothelial hyaluronan receptor (LYVE-1) also show altered expression during active RA, suggesting their utility as biomarkers for monitoring disease activity (70). Focusing on cell-type-specific EVs, Huang et al. identified upregulation of dihydropyrimidinase like 3 (DPYSL3) and downregulation of proteasome activator subunit 1 (PSME1) in CD4^+^ T cell-derived EVs, pointing to their promise as novel RA-specific biomarkers (71). Collectively, multiple proteins have been identified in circulating EVs as promising biomarkers for the diagnosis, disease activity monitoring, and treatment response prediction in RA.

Beyond exosomal cargo, the quantitative distribution of different EV subtypes can also reflect disease progression. Michael et al. observed elevated levels of Annexin V^+^ MPs, along with MPs derived from platelets and leukocytes, highlighting their potential as diagnostic markers (61). In another study, Arntz et al. demonstrated that RA patients with IgM-RF present on plasma EV surfaces showed higher disease activity, indicating that such vesicle subpopulations may be useful for evaluating disease severity and predicting therapeutic response (72).

## EV-based therapy development for RA

In addition to their established roles in intercellular communication and as diagnostic biomarkers, EVs are increasingly recognized as promising candidates for therapeutic development. This potential is realized through two main approaches. The first involves the direct use of naturally secreted EVs as therapeutic agents, leveraging bioactivity inherited from parent cells to regulate recipient cell functions (73). The second centers on engineering EVs into efficient drug delivery systems that can load and transport compounds (74). In RA, a growing body of evidence highlights the therapeutic promise of EVs derived from various sources. By modulating RA-relevant cells, these EVs can slow disease progression and delivering measurable therapeutic benefits.

### Natural EVs for RA therapy

**Figure 2:**
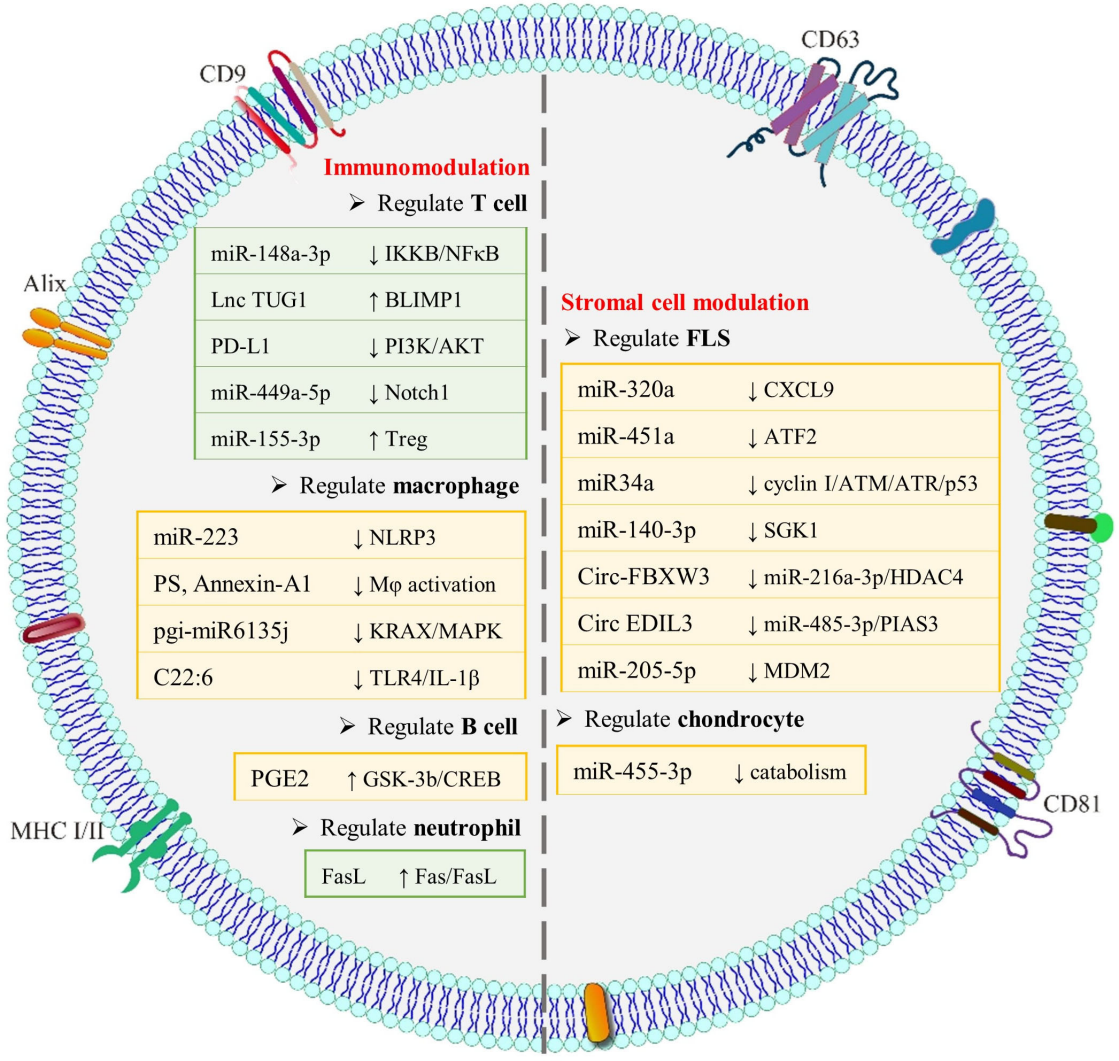
Roles and mechanisms of exosomal RNAs, proteins and lipids from natural EVs in RA therapy. These bioactive molecules modulate FLS, endothelial cells, chondrocytes and immune cells. By acting on joint stromal cells and rebalancing immune responses, they delay, block or even reverse RA progression.

In RA treatment, core strategies involve immunomodulation and the reprogramming of joint stromal cells. EVs derived from various sources, such as MSCs, immune cells, plants, and bacteria, naturally possess therapeutic potential in RA. This potential arises from their natural cargo, including nucleic acids, proteins and lipids, which enables them to engage these key therapeutic mechanisms. **Figure 2** summarizes the principal cargoes of EVs through which EVs exert immunomodulatory effects or regulate stromal cell functions in the context of RA treatment.

#### Immunomodulation

In RA, immune dysfunction is a central driver of disease. Persistent immune cell activation promotes excessive pro-inflammatory cytokine release and autoantibody production, leading to synovial hyperplasia, inflammatory infiltration, and erosion of cartilage and bone. Current first-line treatments, the disease-modifying anti-rheumatic drugs (DMARDs), are predominantly immunosuppressive. While effective for symptom control, these agents have limitations in long-term safety, immune tolerance induction, and tissue repair. Emerging immunomodulatory strategies offer a promising alternative by not only suppressing inflammation but also promoting immune balance. EVs from specific sources exhibit intrinsic immunomodulatory activity and can regulate diverse immune cells to remodel the immune environment.

T cell dysregulation is a central driver of immune dysfunction in RA. Various EVs have been implicated in regulating T cell activity in this context. For example, Xu et al. showed that EVs derived from umbilical cord MSCs (UCMSC-EVs) suppress T cell proliferation, promote T cell apoptosis, and modulate the Th17/Treg balance (75). Notably, high-dose EVs outperformed MTX in improving the arthritis index (assessed by joint hyperemia, inflammation and swelling), suggesting a potentially superior therapeutic effect on specific immune parameters. The regulatory functions of EVs on T cells are largely mediated by their bioactive RNA and protein cargo. One study reported that EVs from gingival MSCs (GMSC-EVs) are enriched with miR-148a-3p, which targets and inhibits the IκB kinase β (IKKB)/NF-κB pathway in T cells (76). These GMSC-EVs have exhibited both inflammatory targeting and therapeutic efficacy in CIA models and humanized models involving inflamed FLS, supporting their potential as a cell-free strategy for RA. Similarly, EVs derived from bone marrow MSCs (BMSC-EVs) carrying Lnc TUG1 (taurine-upregulated gene 1) were shown to delay RA progression by modulating T cell activity (77). Following delivery into CD4^+^ T cells, Lnc TUG1 upregulates the transcription factor B-lymphocyte-induced maturation protein-1 (BLIMP1), thereby promoting Treg differentiation while suppressing Th17 polarization and rebalancing the Th17/Treg axis. Rui et al. reported that EVs derived from olfactory ecto-MSCs (OE-MSC-EVs) highly enriched with the immune checkpoint protein PD-L1, which markedly suppresses Tfh cell polarization through the phosphatidylinositol 3- kinase (PI3K)/AKT pathway. This suppression consequently reduced germinal center B cell and plasma cell differentiation and attenuated autoimmune responses. Beyond MSC-EVs, EVs derived from immune cells also regulate T cells in RA. Tregs, essential for maintaining immune tolerance, release EVs (Treg-EVs) with therapeutic potential. Chen et al. demonstrated that transforming growth factor β (TGF-β)-induced Treg-EVs specifically home to inflamed joints (78). By delivering highly expressed miR-449a-5p, these EVs suppress the Notch1 pathway, thereby restoring the Th17/Treg balance *in vitro* and in a CIA model. Tolerogenic dentritic cells (tolDCs), characterized by reduced inflammatory responsiveness after agonist exposure, represent another EV source (79). Lin et al. generated microvesicle mimetics (MVM) via extrusion of tolDCs (80). These MVM promoted Treg proliferation and M2 macrophage polarization. Mechanistically, miR-155-3p enriched in MVM inhibits suppressor of cytokine signaling 1 (SOCS1), potentiates interleukin-2 receptor (IL-2R)/signal transducer and activator of transcription 5 (STAT5) signaling, upregulates forkhead box protein P3 (Foxp3) expression, and enhances Treg differentiation, thereby establishing a robust tolerogenic immune environment. Together, these studies underscore the potential of MSC-EVs and immune cell-derived EVs as sophisticated biological tools capable of reprogramming T cell dysfunction for RA treatment.

Macrophages play critical roles in RA pathogenesis, characterized by excessive cytokine release and disrupted M1/M2 balance. Modulating these functions through EVs offers a promising strategy for attenuating disease progression. MSC-EVs are widely recognized for their immunomodulatory effects on macrophages. For example, Zhang et al. demonstrated that EVs from embryonic stem cell-derived MSCs (ESC-MSC-EVs) promote M2 polarization while reducing M1 macrophage infiltration in synovium tissues, thereby restoring macrophage homeostasis (81). Similarly, BMSC-EV-delivered miR-223 was shown to suppress NLR family pyrin domain-containing 3 (NLRP3) inflammasome activation in macrophages, reducing secretion of IL-1β and IL-1, and exerting significant anti-inflammatory effects both *in vitro* and in rat models (82). Immune cell-derived EVs also exhibit potent macrophage-modulating activity. Kim et al. reported that EVs from M2 macrophage (M2-EVs) can reprogram synovial M1 macrophages toward an M2-like phenotype (83). Following intra-articular injection in a CIA murine model, M2-EVs persisted in the joints for more than three days, significantly ameliorating joint swelling, inflammation scores, and bone erosion, with efficacy comparable to MTX. In another study, Rhys et al. found that neutrophils stimulated with TNF-α release MVs (NDMVs) that suppress lipopolysaccharide (LPS)/interferon-γ (IFN-γ)-induced pro-inflammatory polarization of macrophages (84). Mechanistically, phosphatidylserine exposed on the NDMVs engages macrophage Mer tyrosine kinase (MerTK) receptor, while annexin A1 activates formyl peptide receptor 2 (FPR2) to promote TGF-β release. Plant-derived EVs represent another promising avenue, offering advantages such as excellent biocompatibility, low immunogenicity, and scalable production. Han et al. engineered folic acid-modified ginger-derived EVs (FA-GDEVs) to target the folate receptor β (FRβ) on M1 macrophage in inflamed joints (85). FA-GDEVs promoted the M1-to-M2 transition through activation of the PI3K/AKT pathway, effectively alleviating joint pathology in CIA mice without systemic toxicity. Separately, Wang et al. elucidated how ginseng-derived EVs (GEVs) deliver a plant-specific microRNA, pgi-miR6135j, to synovial macrophages (86). This miRNA targets the 3’ untranslated region of Kirsten rat sarcoma viral oncogene homolog (KRAS) mRNA, inhibiting MAPK pathway activation and subsequent IL-6 and TNF-α release, thereby reducing joint inflammation and bone erosion. Brown adipocyte-derived EVs have also demonstrated immunomodulatory potential. One study revealed that docosahexaenoic acid (C22:6) carried by these EVs binds directly to toll-like receptor 4 (TLR4) on macrophages, disrupting IL-1β signaling and conferring protective effects against RA (87). Together, these findings underscore how EVs from diverse sources can precisely modulate macrophage behavior, opening new avenues for targeted and comprehensive RA therapy.

Natural EVs derived from specific cell types can also modulate the pathogenic functions of B cells in RA. For example, Cosenza et al. reported that BMSC-MVs reduce plasmablast differentiation, an effect potentially mediated by EV cargo such as TGF-β1, prostaglandin E2 (PGE2), and IL1 receptor antagonist (IL1RA) (88). Beyond MSCs, granulocytic myeloid-derived suppressor cells (G-MDSCs), which play a key immunomodulatory role in suppressing autoimmune pathology, also serve as an effective cellular source of EVs that regulate B cell activity. Studies have shown that EVs derived from G-MDSCs (G-EVs) deliver PGE2, which induces phosphorylation of glycogen synthase kinase-3 β (GSK-3β) and cyclic AMP response element binding (CREB), thereby promoting the differentiation of interleukin 10 (IL-10)^+^ regulatory B cells (89). This shift was associated with decreased proportions of Tfh cells and plasma cells, lower levels of total serum IgG and collagen-specific antibodies, and ultimately alleviation of joint inflammation and pathological damage *in vivo*. In summary, natural EVs from BMSCs and G-MDSCs represent promising cell-free therapeutics for RA by targeting B cell dysfunction.

In RA, neutrophils are recruited to the synovial joints, where they contribute to disease progression through mechanisms such as the release of inflammatory mediators and the formation of neutrophil extracellular traps. Consequently, strategies aimed at modulating neutrophil activity represent a promising therapeutic approach for RA management. To this end, Kang et al. developed a multifunctional nanomaterial designated as D@ApoEVFasL∩L, constructed by loading dexamethasone and heparin (modified with a reactive oxygen species (ROS)-responsive linker onto BMSC-ApoBDs) (90). These BMSC-ApoBDs exhibit high presentation of Fas ligand (FasL). The system specifically targets inflamed joints, where the conjugated heparin competitively inhibits P-selectin, thereby blocking neutrophil recruitment. Under high ROS conditions, the material releases heparin and exposes FasL, triggering neutrophil apoptosis through the Fas/FasL signaling pathway. The resulting apoptotic cells further promote macrophage polarization toward the M2 phenotype, while the concurrently released dexamethasone scavenges ROS, exerting a synergistic therapeutic effect.

#### Stromal cell modulation

In RA, stromal cells within the joint undergo substantial functional alterations. Correcting these dysregulated cellular states represents a promising therapeutic strategy. Recent studies indicate that EVs derived from certain cell types can promote the restoration of normal cellular function by modulating key signaling pathways in stromal cells, highlighting their potential as a novel therapeutic approach for RA.

FLS serve as pivotal stromal effector cells in RA, playing a central role in disease pathogenesis. Their dysregulated behavior, characterized by hyperproliferation, apoptosis resistance, aggressive migration, excessive secretion of inflammatory cytokines, and promotion of angiogenesis, directly drives synovitis and joint destruction. Consequently, targeting FLS represents a rational therapeutic strategy in RA. In this context, MSC-EVs have emerged as a promising cell-free platform capable of reprogramming RA-FLS pathology through the delivery of regulatory molecular cargoes. For example, Meng et al. demonstrated that BMSC-EVs deliver miR-320a to RA-FLS, effectively suppressing their activation, migration, and invasion by downregulating CXCL9 expression, thereby alleviating arthritis symptoms in a mouse model (91). Similarly, UCMSC-EVs deliver miR-451a, which targets activating transcription factor 2 (ATF2), leading to the inhibition of FLS proliferation, migration, and invasion (92). Beyond modulating growth and invasive behavior, MSC-EVs effectively attenuate the inflammatory function of FLS. BMSC-EVs carrying miR-34a inhibit inflammatory factor secretion in FLS by suppressing Cyclin I and activating the p53 pathway (93). Similarly, UCMSC-EV-delivered miR-140-3p targets serum- and glucocorticoid-inducible kinase 1 (SGK1), reducing inflammatory cytokine expression in RA-FLS (94). Furthermore, MSC-EVs regulate RA-FLS via circRNAs. BMSC-EV-derived circFBXW7 acts as a molecular sponge for miR-216a-3p, thereby alleviating its repression of histone deacetylase 4 (HDAC4). This mechanism inhibits FLS proliferation, migration, invasion and the secretion of pro-inflammatory factors (82). Additionally, MSC-EVs modulates RA-FLS to inhibit angiogenesis. Zhang et al. demonstrated that synovial MSC-derived EVs (SMSC-EVs) deliver circEDIL3, which functions as a molecular sponge for miR-485-3p (95). This interaction relieves miRNA-mediated suppression of protein inhibitor of activated STAT 3 (PIAS3), thereby inhibiting STAT3 activation and the expression of its downstream target vascular endothelial growth factor (VEGF). This ultimately suppresses inflammation-induced angiogenesis and alleviates RA progression. Notably, strategic preconditioning of MSCs can enhance EV efficacy. For instance, chondrogenically-induced BMSC-EVs enriched with miR-205-5p suppress MAPK and NF-κB pathways in FLS through the MDM2 axis, reducing inflammatory mediators and matrix metalloproteinases (MMPs) (96). Collectively, these findings establish MSC-EVs as versatile therapeutics capable of normalizing the aggressive phenotype of RA-FLS through simultaneous targeting of multiple pathogenic pathways, offering a novel and promising strategy to intercept RA progression.

In the pathogenesis of RA, chondrocytes represent primary targets within the progressively deteriorating articular microenvironment. Disruption of their cellular homeostasis leads to pathological phenotypic alterations, ultimately driving cartilage degradation. Studies have shown that natural EVs can modulate chondrocyte pathology and alleviate disease manifestations. For example, Wang et al. reported that BMSC-EVs are enriched with ECM and adhesion-related proteins (97). These EVs can be internalized by chondrocytes, promoting their proliferation and migration *in vitro*. In a rat model of RA, administration of such EVs significantly alleviated joint swelling, suppressed synovial inflammation, and promoted cartilage repair. Another study highlighted the therapeutic potential of neutrophil-derived EVs (NDEVs). Thomas et al. demonstrated that a single intra-articular injection of NDEVs significantly attenuated proteoglycan loss and structural cartilage damage (98). Mechanistic investigations revealed that miR-455-3p plays a central role in mediating the anti-catabolic and pro-regenerative effects of NDEVs on chondrocytes via modulation of Wnt and Notch signaling pathways. Notably, NDEVs derived from RA patients exhibited similarities to those from healthy donors in terms of size, quantity, miR-455-3p content, and cartilage-protective function, suggesting their potential as an autologous therapeutic source for RA treatment. Together, these findings underscore the therapeutic potential of EVs in mitigating chondrocyte dysfunction and promoting cartilage repair in RA.

In RA, the abnormally elevated number of mature OCs constitutes the direct cellular driver of peri-articular bone erosion; consequently, pharmacological inhibition of OC formation represents a rational strategy to retard erosive progression. Li et al. reported that ApoBDs derived from macrophages (Mφ-ApoBDs) suppress OC maturation and confer therapeutic efficacy in RA animal models (99). Additionally, outer membrane vesicles (OMVs) secreted by Propionibacterium freudenreichii MJ2 (a Gram-positive, food-grade bacterium widely used in cheese production) were shown to mitigate joint bone erosion by blocking the RANK–RANKL interaction (100). Concurrently, these OMVs modulate inflammatory cytokine balance, collectively leading to significant amelioration of arthritis symptoms in animal models.

### Engineered EVs as drug delivery vehicles for RA therapy

EVs represent a highly promising class of drug delivery platforms for RA therapy. They can be precisely engineered to encapsulate diverse therapeutic agents, including small molecules, nucleic acids, proteins, and nanomaterials. Here, we summarizes recent advances in the development and application of EV-based delivery systems for RA treatment.

#### EVs as delivery vehicles for small molecules

Small-molecule drugs offer considerable advantages in drug development, characterized by high oral bioavailability and the ability to target intracellular proteins. However, many candidate molecules are limited by poor water solubility or low stability. EVs represent a promising strategy to enhance the clinical applicability of small-molecule drugs by improving their solubility and stability, which facilitates better aqueous dispersibility and preservation of bioactivity (101). Moreover, by harnessing the innate targeting properties of EVs or applying targeted modifications, precise drug delivery can be achieved, thereby enhancing drug utilization and therapeutic efficacy. In the context of RA, encapsulating small molecules with immunosuppressive, anti-inflammatory, and anti-oxidative properties into EVs has been demonstrated to substantially improve therapeutic outcomes (**Table 2**).

**Table 2:** EV-delivered small molecule drugs for RA treatment.

Small molecule	EV vehicle	Loading manner	Targeting ability	Ref
MTX	osteosarcoma cell-derived EV	Sonication	Native bone tissue targeting ability inherited from parental cells	(103)
MTX	UCMSC-EV	Cell incubation	Anti-CD80 modification to target CD80+ macrophages	(104)
Triptolide	RA-FLS-EV	Electroporation	–	(106)
Triptolide	DC-EV	Incubation	Native DC targeting ability inherited from parental cells	(107)
Rapamycin	M2-EV	sonication	Native inflammatory tissue targeting ability inherited from parental cells	(109)
Dexamethasone	Mφ-EV	Electroporation	Folic acid modification to target the inflamed joint	(111)
Dexamethasone	BMSC-apoBD	Cell incubation	Native inflamed joint targeting ability inherited from parental cells	(90)
Betamethasone sodium phosphate	M2-EV	Electroporation	Native inflamed joint targeting ability inherited from parental cells	(112)
Curcumin	ADSC-EV	Incubation	–	(114)
EGCG	Mφ-EV	Sonication	–	(115)
Icariin	ADSC-EV	Incubation	–	(116)
Purple sweet potato anthocyanins	RA-FLS-EV	Electroporation	Native inflammatory cell targeting ability inherited from parental cells	(117)
Phenylpropanoids	Mφ-EV	Sonication & Incubation	–	(118)

MTX, a first-line immunosuppressant for RA, possesses anti-inflammatory and immunomodulatory properties (102). However, its clinical application is constrained by poor water solubility, low bioavailability, and non-specific tissue distribution, which contribute to systemic adverse effects such as oral mucosal injury, hepatotoxicity, and myelosuppression (102). Therefore, the development of targeted delivery systems that enhance local drug concentration while minimizing systemic exposure is crucial to optimizing the therapeutic profile of MTX. Zhang et al. utilized the inherent bone-targeting capability of osteosarcoma cell-derived EVs to develop a MTX delivery platform (103). By encapsulating MTX-loaded EVs within a HA–chondroitin sulfate hydrogel, the composite system enabled sustained drug release and reduced the frequency of systemic administration following intra-articular injection. In a CIA rat model, this system effectively scavenged ROS, promoted M2 macrophage polarization, suppressed inflammatory responses, and facilitated cartilage repair, demonstrating comprehensive therapeutic efficacy. In another study, Yang et al. engineered UCMSC-EV decorated with surface CD80 antibodies to target CD80^+^ macrophages for MTX delivery (104). Following cellular uptake, MTX release induced a shift toward an anti-inflammatory macrophage phenotype and upregulated TGF-β expression. This process promoted Treg differentiation while suppressing T helper 1 (Th1) cell activation. In the CIA model, the engineered EVs markedly alleviated joint swelling, reduced inflammatory cytokine levels, and restored immune homeostasis, illustrating an innovative and efficient targeted strategy for MTX delivery. Thus, both natural and engineered EVs serve as promising vehicles for targeted MTX therapy in RA.

Triptolide, a diterpenoid epoxide derived from *Tripterygium wilfordii*, exhibits potent anti-rheumatic and immunomodulatory effects, demonstrating efficacy in alleviating RA pathology (105). However, its clinical application is limited by poor aqueous solubility, a narrow therapeutic window, and significant systemic toxicity. To overcome these challenges, Jiang et al. utilized the natural joint-targeting capability of EVs derived from RA-FLS to construct a triptolide delivery platform (106). The EV-based system displayed improved articular retention, enabling sustained drug release at the injection site for over 24 hours. In a CIA mouse model, this formulation significantly attenuated joint swelling and inflammatory cell infiltration. In addition to FLS-EVs, EVs originating from immune cells offer inherent targeting advantages owing to the surface antigens inherited from their parental cells. For example, Rao et al. designed a delivery system using DC-derived EVs to transport triptolide (107). These EVs effectively delivered the payload to DCs *in vivo*, resulting in marked suppression DC activation and modulation of T cell differentiation. The strategy produced pronounced anti-inflammatory and therapeutic outcomes in an RA model. Furthermore, its selective targeting capability reduced triptolide accumulation and associated toxicity in organs such as the liver, thereby enhancing treatment safety.

Rapamycin, an mTOR inhibitor, contributes to immune tolerance in RA (108). However, its systemic administration lacks tissue specificity and can lead to off-target effects. To address this limitation, Wu et al. designed a multifunctional nanocomposite termed M2CPR, constructed by loading rapamycin, copper sulfide nanoparticles (CuS NPs), and CitP to M2-EVs (109). The M2-EVs enabled targeted delivery to inflamed joints in RA. Upon arrival, released CuS NPs triggered cuproptosis in activated T cells. The resulting cellular fragments were engulfed by macrophages, leading to abundant secretion of TGF-β. TGF-β acted synergistically with the co-delivered rapamycin to induce the differentiation of immature DCs into tolDCs. These tolDCs then present CitP to naive T cells, further promoting Treg differentiation. The newly generated Tregs, in turn, produce additional TGF-β, thereby inducing tolDC formation and establishing a self-amplifying cycle of immune tolerance. This cascade positions M2CPR as a new paradigm for RA management.

Glucocorticoids are widely used in the clinical management of RA owing to their potent anti-inflammatory effects and their ability to rapidly alleviate joint swelling and pain (110). Targeted delivery of glucocorticoids via EVs may prevent drug distribution in non-target tissues, thereby minimizing the risk of adverse effects such as immunosuppression. For example, Yan et al. employed folic acid-modified Mφ-EV to achieve targeted delivery of dexamethasone (111). This formulation significantly enhanced dexamethasone accumulation in inflamed joints through folate receptor-mediated active targeting, effectively alleviating joint swelling and bone erosion and providing a novel strategy for precision glucocorticoid therapy. In another study, researchers loaded dexamethasone into multiply modified BMSC-apoBD (90). The composite system enabled targeted delivery of dexamethasone to inflamed joints, remodeled the RA inflammatory microenvironment, and produced synergistic therapeutic effects by modulating the neutrophil–macrophage–ROS pathogenic axis. Similarly, Li et al. utilized M2-EVs to co-deliver plasmid DNA (pDNA) encoding IL-10 and betamethasone sodium phosphate (112). Owing to integrins present on the surface of M2-EVs, the system specifically targeted inflamed synovium, demonstrating synergistic anti-inflammatory activity and effectively promoting macrophage polarization from the pro-inflammatory M1 to the anti-inflammatory M2 phenotype, which reduced joint swelling and cartilage damage. In summary, EV-mediated targeted delivery of glucocorticoids enhances drug retention at disease sites, allowing immunomodulatory actions to be exerted while reducing systemic side effects. This approach offers a promising new avenue for achieving safer glucocorticoid therapy in RA.

Beyond glucocorticoids, a wide range of natural products have attracted considerable interest for the treatment of RA due to their anti-inflammatory and antioxidant properties (113). However, these molecules often suffer from poor water solubility, low chemical stability, inadequate oral bioavailability, and rapid *in vivo* metabolism. EVs can serve as delivery vehicles to improve both their physicochemical properties and targeting capability. For instance, He et al. employed EVs derived from adipose tissue-derived stem cells (ADSC-EVs) to deliver curcumin, which not only improved the solubility, stability, and sustained-release profile of curcumin, but also demonstrated marked anti-inflammatory and anti-synovial hyperplasia effects in LPS-induced synovial cells (114). Similarly, Song et al. utilized Mφ-EVs to deliver epigallocatechin-3-gallate (EGCG), thereby enhancing its stability and sustained-release behavior while improving targeted delivery to chondrocytes (115). In a rat arthritis model, this system significantly alleviated joint swelling, synovial hyperplasia, and cartilage structural damage. In another study, Yan et al. utilized ADSC-EVs to deliver icariin, which effectively accumulated in the joints of arthritic rats and alleviated inflammation and tissue damage by reprogramming macrophage metabolism (116). Additionally, Dong et al. encapsulated purple sweet potato anthocyanins within RA-FLS-EVs, creating a delivery system with inflammatory targeting capability (117). Experimental results demonstrated that this system could be efficiently recognized and internalized by inflammatory cells, substantially enhancing cellular uptake and anti-inflammatory activity of anthocyanins. Patel et al. also delivered phenylpropanoid via Mφ-EVs, improving its stability and exhibiting notable anti-inflammatory effects *in vitro* (118). In summary, EVs serve as excellent delivery vehicles for natural anti-inflammatory molecules, enhancing their stability and targeting precision, and holding substantial promise for RA therapy.

#### EVs as delivery vehicles for nuclei acids

Nucleic acid therapeutics offer revolutionary potential through their ability to directly encode functional proteins or precisely regulate gene expression. However, their clinical efficacy is limited by poor membrane permeability, which stems from their inherent negative charge and susceptibility to nuclease-mediated degradation. EVs represent promising natural delivery vehicles for nucleic acids. Engineered EVs, loaded with therapeutic nucleic acids, have demonstrated significant potential for the treatment of RA (**Table 3**) (119).

**Table 3:** EV-delivered nucleic acids for RA treatment.

Nucleic acid	EV vehicle	Loading manner	Targeting ability	Ref
miR-146a & miR-155	ADSC-EV	Cell transduction	–	(121)
miR-146a	ADSC-EV	Cell transduction	–	(122)
miR-10a	BMSC-EV	Cell transduction	–	(123)
miR-150-5p	BMSC-EV	Cell transduction	–	(124)
miR-124a	BMSC-EV	Cell transduction	–	(126)
miR-150-5p	UCMSC-EV	Cell transduction	–	(125)
miR-433-3p	SMSC-EV	Cell transduction	–	(127)
miR-486-5p	RA-FLS-EV	Cell transduction	–	(128)
TNF-α siRNA	milk-EVs	Sonication	Microneedle allowed local administration	(131)
IL-10 expressing plasmid	M2-EV	Electroporation	Native inflamed joint targeting ability inherited from parental cells	(112)

miRNAs are short non-coding RNAs that post-transcriptionally fine-tune gene expression through mRNA degradation or translational repression. Their capacity to simultaneously modulate multiple targets has attracted significant interest for therapeutic applications in various diseases (120). Encapsulation within EVs protects miRNAs from rapid degradation in circulation and enables targeted tissue accumulation via surface engineering. Consequently, EVs from diverse sources have been explored for delivering therapeutic miRNAs in RA. For example, studies have shown that MSC-EVs can deliver multiple miRNAs, including miR-146a (121, 122), miR-155 (121), miR-10a (123), miR-150-5p (124, 125), miR-124a (126), and miR-433-3p (127), for RA therapy (**Table 3**). Notably, MSC-EVs function not merely as passive carriers but also exert intrinsic immunomodulatory effects, thereby synergistically contributing to RA amelioration. As an example, Wang et al. loaded miR-150-5p into UCMSC-EVs and demonstrated in a RA model that these EVs activated the aryl hydrocarbon receptor pathway, leading to suppressed OC differentiation, enhanced Treg cell proliferation and function, alleviated bone destruction, and restored osteoimmune balance (125). Beyond MSC-EVs, EVs from other cellular sources have also been harnessed for miRNA delivery. For example, RA-FLS-EVs were employed to deliver miR-486-5p, which targeted and downregulated the transducer of erbB-1 (Tob1) gene in osteoblasts (128). This modulation activated the bone morphogenetic protein (BMP)/Smad pathway, promoted osteoblast differentiation, and mitigated bone erosion in RA.

Small interfering RNAs (siRNAs), capable of specifically silencing disease-causing genes, hold significant potential for treating chronic inflammatory diseases such as RA (129). However, they exhibit poor plasma stability and limited cellular uptake. Milk-derived EVs (mEVs) have emerged as an attractive nucleic acid delivery vector owing to their wide availability, high yield, excellent biocompatibility, and ease of isolation (130). In a study by Wen et al., TNF-α siRNA was loaded into mEVs via ultrasonication and incorporated into a frozen microneedle patch (131). Transdermal administration at acupoints effectively alleviated arthritic symptoms in a rabbit RA model. This microneedle system markedly suppressed synovial cell proliferation, lowered pro-inflammatory cytokine levels, and restored the MMP/tissue inhibitor of metalloproteinases-1 (TIMP-1) balance, with acupoint application yielding superior therapeutic outcomes compared to non-acupoint application.

pDNA, a pivotal tool in gene therapy, expands the therapeutic repertoire by enabling sustained expression of therapeutic proteins (132). EVs again offer pharmacological advantages for pDNA delivery. In a study by Li et al., M2-EVs were employed as carriers to co-load IL-10-encoding pDNA along with a glucocorticoid drug (112). This combined system demonstrated notable anti-inflammatory efficacy both *in vitro* and *in vivo*, demonstrating considerable potential for RA treatment.

#### EV as delivery vehicles for proteins

The clinical translation of protein-based therapeutics is often limited by inherent challenges, including poor cellular permeability, susceptibility to proteolytic degradation, and potential immunogenicity. EVs have emerged as promising vehicles for protein therapeutics, as they can protect cargo from degradation, enhance cellular uptake, and enable targeted delivery (133). In RA, EVs have been utilized to deliver diverse immunomodulatory and anti-inflammatory proteins, demonstrating considerable potential in mitigating disease progression (**Table 4**).

**Table 4:** EV-delivered proteins for RA treatment.

Protein	EV vehicle	Loading manner	Targeting ability	Ref
JKAP	BMSC-EV	Cell transduction	–	(134)
FGL1	BMSC-EV	Cell transduction	–	(135)
CTLA4Ig	iMSC-EV	Cell transduction	–	(136)
IL-10	Mφ-EV	Electroporation	Native inflammation targeting ability inherited from parental cells; Ultrasound targeting	(137)
IL-10 & Anti-TNF	NDEV	Sonication & Lipid fusion	Anti-ROS-CII modification to target damaged cartilage	(98)
IL-4	293T-EV	Cell transduction	–	(139)
srIκBα	293T-EV	Cell transduction	–	(140)

MSC-EVs have been extensively investigated as delivery vehicles for therapeutic proteins in RA. Their inherent immunomodulatory and tissue-reparative properties can act synergistically with the encapsulated therapeutic protein cargo to alleviate RA pathogenesis. For example, Xu et al. demonstrated that BMSC-EVs delivering JNK pathway-associated phosphatase (JKAP) to CD4+ T cells suppressed the AKT/extracellular regulated protein kinases (ERK) pathway, thereby restoring Th17/Treg balance (134). Another study showed that BMSC-EVs loaded with fibrinogen-like 1 (FGL1) promoted FLS apoptosis and inhibited cytokine release via suppression of the NF-κB pathway, contributing to immunoregulation (135). Similarly, Choi et al. utilized EVs from immortalized MSCs (iMSCs) to deliver cytotoxic T-lymphocyte antigen 4 immunoglobulin (CTLA4Ig), which increased T helper 2 (Th2) cell populations, reduced plasma cell and macrophage infiltration, and modulated key cytokines, leading to enhanced therapeutic efficacy compared to unmodified EVs in an animal model (136). Collectively, these findings suggest that MSC-EV-based protein delivery represent a dual-targeting strategy for RA therapy.

Similar to MSC-EVs, immune cell-derived EVs have also emerged as promising platforms for protein delivery in RA, owing to their intrinsic immunomodulatory functions that can confer additional therapeutic benefits. For instance, Tang et al. utilized Mφ-EV to deliver IL-10, with ultrasound assistance enhancing joint-specific targeting. This approach effectively polarized macrophages toward the M2 phenotype, reduced inflammation, and promoted tissue repair (137). In another approach, Topping’s team engineered NDEVs modified with anti-ROS-CII antibody to co-deliver IL-10 and an anti-TNF agent (98). This system enabled precise targeting of damaged cartilage and markedly accelerated the resolution of synovial inflammation.

The 293T cell line, a well-established model in biomedical research for its high transfection efficiency and ease of genetic modification, has been widely utilized for producing engineered EVs (138). For example, Takenaka et al. effectively loaded IL-4 onto EVs by fusing it with lactadherin, which facilitated macrophage-specific targeting and induced M2 polarization. This approach demonstrated superior efficacy and safety compared to free IL-4 in an arthritis model (139). Similarly, Lee’s team engineered 293T cells to secrete EVs carrying a super-repressor IkB (srIκB) protein, which significantly suppressed inflammatory cytokine release and alleviated joint pathology (140).

#### EV as delivery vehicles for nanomaterials

In recent years, novel nanomaterials have demonstrated considerable therapeutic potential across various diseases (141). However, their clinical translation remains challenging due to several limitations, such as insufficient physiological stability, lack of targeting specificity, and uncertainties regarding long-term biosafety. To address these challenges, EVs have been employed to encapsulate and protect nanomaterials, thereby enhancing their stability and site-specific accumulation. This integrated strategy facilitates safer and more precise therapeutic delivery, offering a promising pathway toward the clinical application of nanomaterial-based therapies (**Table 5**).

**Table 5:** EV-delivered nanomaterials for RA treatment.

Nanomaterial	EV vehicle	Loading manner	Targeting ability	Ref
uPB nanozyme	NDEV	Chemical ligation	Native inflamed joint targeting ability inherited from parental cells	(143)
Ru-HFO	BMSC-EV	Sonication	Targeted acting upon ultrasound stimulation	(144)
BP	M2-extruded NV fused with M1-cell membrane	Electroporation	Native inflamed joint targeting ability inherited from parental cells	(146)
CuS NPs	M2-EV	Sonication	Native inflammatory tissue targeting ability inherited from parental cells	(109)
MTX-encapsulated mesoporous silica nanoparticles	ADSC-EV	Sonication	Native inflamed joint targeting ability inherited from parental cells	(147)

Nanozymes are catalytic nanomaterials that mimic enzymatic functions to restore intracellular redox balance, offering considerable potential for RA treatment (142). EV-mediated delivery of nanozymes can enhance their targeting specificity and reduce systemic clearance. For example, Zhang et al. covalently conjugated ultrasmall Prussian blue nanoparticles (uPB) to the surface of NDEVs via copper-free click chemistry (143). The resulting engineered EVs inherited targeting molecules from their parent cells, enabling specific recognition and accumulation within inflamed RA joints and subsequent penetration into cartilage tissue. These EVs effectively neutralized pro-inflammatory factors, scavenged ROS, alleviated inflammatory stress, and restored immune balance by modulating the Th17/Treg axis, thereby significantly ameliorating joint damage. In another study, Xiao et al. utilized BMSC-EVs to deliver Ru cluster-anchored hydroxylated Fe_2_O_3_ (Ru-HFO) in combination with ultrasound stimulation (144). This approach effectively scavenged ROS, alleviated hypoxia, promoted macrophage repolarization from the M1 to the M2 phenotype, and suppressed osteoclastogenesis, collectively reshaping the joint immune microenvironment and restoring articular function.

Black phosphorus (BP) exhibits outstanding physicochemical properties for photothermal therapy (145). Nevertheless, its biomedical application remains constrained by rapid hydrolysis, susceptibility to surface oxidation, and an insufficient long-term safety profile. Encapsulation within EVs can stabilize BP nanosheets, delay their degradation, and improve biocompatibility. Zhao et al. developed a hybrid EV-mimetic nanovesicle system by fusing M2-EVs with M1 macrophage membranes to encapsulate BP nanosheets (146). This system not only neutralized inflammatory cytokines and released anti-inflammatory mediators, but also enabled targeted ablation of inflammatory cells under near-infrared irradiation, resulting in synergistic anti-inflammatory and therapeutic efficacy.

EVs have also been utilized to deliver other functional nanomaterials for RA therapy. For instance, M2-EVs were employed to co-deliver CuS NPs along with other therapeutic agents (109). The engineered EVs selectively accumulated at inflamed joint sites due to the intrinsic targeting ability of M2-EVs. At these locations, CuS NPs triggered cuproptosis in T cells and, in combination with other bioactive cargo within the EVs, induced a cascade of immunomodulatory responses that ultimately promoted immune tolerance. In another strategy, Zhu et al. engineered ADSC-EVs to encapsulate MTX-loaded mesoporous silica nanoparticles, forming a core–shell nanocomposite (147). This formulation demonstrated favorable biocompatibility, sustained drug release, and efficient cellular uptake. It promoted M2 macrophage polarization and inhibited the migration and invasion of FLS. *In vivo*, the nanocomposite specifically accumulated in inflamed joints, significantly alleviating joint swelling, synovial hyperplasia, and bone and cartilage destruction.

## Conclusion

EVs have emerged as pivotal mediators in the pathogenesis, diagnosis, and treatment of RA. They facilitate essential intercellular communication among synovial stromal cells, immune cells, and relevant biofluids. EVs isolated from synovial fluid and peripheral blood show considerable promise as carriers of disease-specific biomarkers. Moreover, EVs derived from various natural sources exhibit therapeutic potential through immunomodulation and the functional reprogramming of joint stromal cells. The development of targeted EV-based drug delivery systems further offers a valuable complement to existing RA therapies. However, most of these advances remain at the foundational research stage, and substantial efforts are needed to bridge the gap toward clinical translation.

As summarized in **Table 1**, numerous studies have identified different EV profiles in the blood and synovial fluid of RA patients, underscoring their diagnostic potential. However, these investigations largely remain descriptive from a basic research perspective. A critical gap persists in systematically validating their diagnostic sensitivity and specificity or in demonstrating clear advantages over established clinical standards. Nevertheless, EVs possess inherent advantages as diagnostic platforms. They encapsulate rich, multidimensional disease information, including biomolecular contents, physical properties, and subtype distributions, which far exceed the informational capacity of traditional soluble biomarkers. Integrating these high-dimensional EV data through advanced analytical tools such as machine learning or digital twin models could inform RA management, enabling robust disease monitoring, guiding personalized treatment strategies, and ultimately supporting dynamic precision medicine in RA (148, 149).

Although preclinical studies strongly support the therapeutic potential of various EVs in RA, no clinical evidence for human RA has been reported. To support this potential, studies on related conditions such as OA and Sjögren’s syndrome have provided preliminarily confirmation of the safety and initial efficacy of MSC-EVs (150, 151). However, several critical challenges must be overcome to advance EVs toward clinical translation. These include scalable purification, stable storage, reliable potency evaluation, efficient drug loading, and appropriate regulatory frameworks. First, regarding purification, a current single-method approach fails to simultaneously achieve scalability and high purity. Evidence suggests that combined strategies, such as integrating tangential flow filtration with size-exclusion chromatography, can enable scalable EV production while maintaining high purity, and thus offer a currently viable technical solution (152). Second, although EVs can be stored long-term at -80°C, repeated freeze-thaw cycles may compromise their bioactivity (153). Moreover, cold-chain transport is costly and logistically challenging. Lyophilization in the presence of suitable cryoprotectants may offer a promising alternative to maintain EV stability during storage and transportation (154, 155). Third, for potency assessment, current methods often depend on specific functional readouts, such as macrophage polarization capacity (156). However, these are insufficient to fully capture the therapeutic potential and quality attributes of EV-based therapeutics. To address this, Izquierdo et al. propose integrating artificial intelligence with multi-omics analysis (covering nucleic acids, proteins, lipids, and metabolites) to build predictive models for real-time monitoring of production parameters and EV characteristics (157). Such an approach could allow early detection of batch inconsistencies or contamination, thereby enhancing process robustness and the comprehensiveness of evaluation. Fourth, in drug loading, conventional techniques such as incubation, electroporation, and sonication often suffer from low efficiency, high cost, and potential structural damage to EVs (158). Developing novel loading strategies that are efficient, gentle, and controllable is therefore essential for advancing EV-based delivery systems. Finally, current regulatory guidelines lack EV-specific standards, leaving existing frameworks inadequate for addressing their unique biological and manufacturing characteristics (159). Establishing EV-tailored quality-control criteria and regulatory pathways will be crucial for promoting standardization and sustainable development in this field.

## Glossary

EVs: extracellular vesicles
MVs: microvesicles
ApoBDs: apoptotic bodies
MSC-EVs: mesenchymal stem cell-derived EVs
RA: rheumatoid arthritis
FLS: fibroblast-like synoviocytes
FLS-EVs: EVs derived from FLSs
RA-FLS-EVs: EVs from RA patient-derived FLS
PDK4: pyruvate dehydrogenase kinase 4
CIAPIN1: cytokine-induced anti-apoptosis inhibitor 1
circRNAs: circular RNAs
lncRNAs: long non-coding RNAs
SOX9: SRY-Box transcription factor 9
CXCL1: chemokine (C-X-C motif) ligand 1
CCR5: C-C chemokine receptor type 5
NF-κB: nuclear factor kappa-B
ECM: extracellular matrix
WTAP: Wilms’
CDO1: cysteine dioxygenase 1
PTX3: pentraxin 3
STAT3: signal transducer and activator of transcription 3
Th17: T helper 17
Tregs: regulatory T cells
HOTTIP: HOXA transcript at the distal tip
Tfh: T follicular helper
CD40L: CD40 ligand
CIA: collagen-induced arthritis
CRKL: CRK like proto-oncogene adaptor protein
ANGPT1: angiopoietin 1
Mφ-EVs: macrophage-derived EVs
mTOR: mammalian target of rapamycin
PBMCs: peripheral blood mononuclear cells
NEAT1: nuclear enriched abundant transcript 1
MDM2: murine double minute-2
SIRT6: sirtuin 6
SF-EVs: synovial fluid-derived EVs
PD-1: programmed death 1
OCs: osteoclasts
TLR7/8: Toll-like receptors 7/8
M-CSF: macrophage colony-stimulating factor
RANKL: receptor activator for nuclear factor-κ
m/lEVs: medium/large extracellular vesicles
IgG: immunoglobulin G
IgM: immunoglobulin M
CPs: citrullinated peptides
IL-1β: interleukin 1β
IL-6: interleukin 6
TNF-α: tumor necrosis factor α
ROCK2: Rho-associated protein kinase 2
DCs: dendritic cells
CitP: citrullinated proteins
CarP: carbamylated proteins
MAPK: mitogen-activated protein kinases
axSpA: axial spondyloarthritis
OA: osteoarthritis
PZP: pregnancy zone protein
HA: hyaluronic acid
HA-EVs: large HA-containing EVs
MPs: microparticles
ACPA: anti-cyclic citrullinated peptide antibodies
RF: rheumatoid factor
sTWEAK: soluble TNF-related weak inducer of apoptosis
anti-CCP: anti-cyclic citrullinated peptide
AUC: area under the curve
mRNAs: messenger RNAs
CCL5: C-C motif chemokine ligand 5
MPIG6B: megakaryocyte and platelet inhibitory receptor G6b
PFKP: phosphofructokinase platelet type
HLA-DR: human leukocyte antigen DR
MTX: methotrexate
AA: amyloid A
LYVE-1: lymphatic vessel endothelial hyaluronan receptor
DPYSL3: dihydropyrimidinase like 3
PSME1: proteasome activator subunit 1
HC: healthy control
DAS28: disease activity score in 28 joints
SDAI: simplified disease activity index
CDAI: clinical disease activity index
VAS: Visual Analogue Scale
ESR: erythrocyte sedimentation rate
DMARDs: disease-modifying anti-rheumatic drugs
UCMSC-EVs: EVs derived from umbilical cord-derived MSCs
GMSC-EVs: EVs from gingival MSCs
IKKB: IκB kinase β
BMSC-EVs: EVs from bone marrow MSCs
TUG1: taurine-upregulated gene 1
BLIMP1: B-lymphocyte-induced maturation protein-1
OE-MSC-EVs: EVs derived from olfactory ecto-MSCs
PI3K: phosphatidylinositol 3- kinase
Treg-EVs: EVs derived from Tregs
TGF-β: transforming growth factor β
tolDCs: tolerogenic dendritic cells
MVM: microvesicle mimetics
SOCS1: suppressor of cytokine signaling 1
IL-2R: potentiates interleukin-2 receptor
STAT5: signal transducer and activator of transcription 5
Foxp3: forkhead box protein P3
ESC-MSC-EVs: EVs derived from embryonic stem cell-derived MSCs
NLRP3: NLR family pyrin domain-containing 3
M2-EVs: M2 macrophage-derived EVs
NDMVs: neutrophil-derived MVs
LPS: lipopolysaccharide
IFN-γ: interferon-γ
MerTK: Mer tyrosine kinase
FPR2: formyl peptide receptor 2
FA-GDEVs: folic acid-modified ginger EVs
FRβ: folate receptor β
GEVs: ginseng-derived EVs
KRAS: Kirsten rat sarcoma viral oncogene homolog
C22:6: docosahexaenoic acid
TLR4: toll-like receptor 4
PGE2: prostaglandin E2
IL1RA: IL1 receptor antagonist
G-MDSCs: granulocytic myeloid-derived suppressor cells
G-EVs: EVs derived from G-MDSCs
GSK-3β: glycogen synthase kinase-3 β
CREB: cyclic AMP response element binding
IL-10: interleukin 10
ROS: reactive oxygen species
FasL: Fas ligand
ATF2: activating transcription factor 2
SGK1: serum- and glucocorticoid-inducible kinase 1
HDAC4: histone deacetylase 4
SMSC-EVs: EVs derived from synovial MSCs
PIAS3: protein inhibitor of activated STAT 3
VEGF: vascular endothelial growth factor
MMPs: matrix metalloproteinases
NDEVs: neutrophil-derived EVs
Mφ-ApoBDs: ApoBDs derived from macrophages
OMVs: outer membrane vesicles
Th1: T helper 1
CuS NPs: copper sulfide nanoparticles
pDNA: plasmid DNA
ADSC-EVs: EVs derived from adipose tissue-derived stem cells
EGCG: epigallocatechin-3-gallate
Tob1: transducer of erbB-1
BMP: bone morphogenetic protein
siRNAs: small interfering RNAs
mEVs: milk-derived EVs
TIMP-1: tissue inhibitor of metalloproteinases-1
JKAP: JNK pathway-associated phosphatase
ERK: extracellular regulated protein kinases
FGL1: fibrinogen-like 1
iMSCs: immortalized MSCs
CTLA4Ig: T-lymphocyte antigen 4 immunoglobulin
Th2: T helper 2
srIκB: super-repressor IkB
uPB: ultrasmall Prussian blue nanoparticles
Ru-HFO: Ru cluster-anchored hydroxylated Fe₂O₃
BP: black phosphorus.
